# DistMLLM: Enhancing Multimodal Large Language Model Serving in Heterogeneous Edge Computing

**DOI:** 10.3390/s25247612

**Published:** 2025-12-15

**Authors:** Xingyu Yuan, Hui Chen, Lei Liu, He Li

**Affiliations:** 1 Department of Sciences and Informatics, Muroran Institute of Technology, Muroran 050-8585, Hokkaido, Japan; 23096502@muroran-it.ac.jp (X.Y.); 23096002@muroran-it.ac.jp (H.C.); 2 Guangzhou Institute of Technology, Xidian University, Guangzhou 510555, China; leiliu@xidian.edu.cn

**Keywords:** edge computing, large language model, task allocation, multi-agent bandit

## Abstract

Multimodal Large Language Models (MLLMs) offer powerful capabilities for processing and generating text, image, and audio data, enabling real-time intelligence in diverse applications. Deploying MLLM services at the edge can reduce transmission latency and enhance responsiveness, but it also introduces significant challenges due to the high computational demands of these models and the heterogeneity of edge devices. In this paper, we propose DistMLLM, a profit-oriented framework that enables efficient MLLM service deployment in heterogeneous edge environments. DistMLLM disaggregates multimodal tasks into encoding and inference stages, assigning them to different devices based on capability. To optimize task allocation under uncertain device conditions and competing provider interests, it employs a multi-agent bandit algorithm that jointly learns and schedules encoder and inference tasks. Extensive simulations demonstrate that DistMLLM consistently achieves higher long-term profit and lower regret than strong baselines, offering a scalable and adaptive solution for edge-based MLLM services.

## 1. Introduction

Large Language Models (LLMs) have emerged as powerful tools capable of understanding and generating human-like responses across a wide range of tasks. Notable examples include OpenAI’s GPT-3 [1], Google’s PaLM [2], and Meta’s LLaMA [3]. While these models have demonstrated remarkable performance on text-based tasks, their capabilities remain largely confined to textual inputs, limiting their effectiveness in scenarios that require processing diverse modalities. To address this limitation, Multimodal Large Language Models (MLLMs) have been developed to integrate and interpret multiple forms of data, such as text, images, and audio. This multimodal capability enables MLLMs to handle complex real-world tasks with a level of sophistication that surpasses traditional LLMs. Recent advancements have driven their adoption in diverse domains such as healthcare [4], autonomous driving [5], and customer service applications, where real-time understanding and response to multimodal inputs are critical.

**Figure 1:**
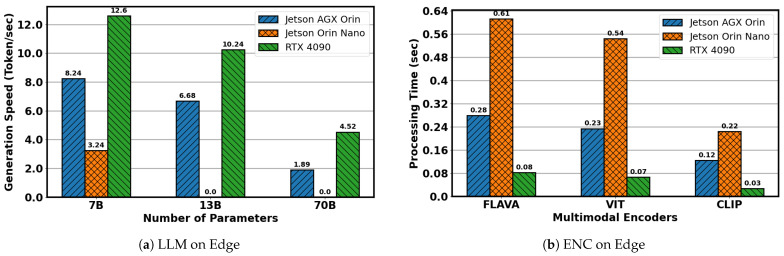
Comparison of different devices for LLM and ENC.

As the demand for low-latency, intelligent services increases, traditional cloud-based solutions face growing limitations. Centralized architectures often incur high latency, bandwidth constraints, and privacy concerns, which can impair the responsiveness of MLLM-powered systems. Deploying MLLMs at the network edge offers a promising alternative [6,7]. However, edge deployment presents significant challenges, primarily due to the heavy computational demands of these models, which often exceed the capabilities of typical edge devices [8,9]. Figure 1a shows the inference performance of three LLaMA models on three representative edge devices. Device specifications are provided in Table 1. Among the tested hardware, the RTX 4090 (NVIDIA, Santa Clara, CA, USA) delivered the highest performance across all model sizes, while the Jetson Orin Nano (NVIDIA, Santa Clara, CA, USA) was unable to complete inference for the 13B and 70B models, underscoring its limited capacity for large-scale deployment. Beyond model inference, multimodal applications also rely on efficient data encoding. To evaluate this aspect, we benchmarked the processing times of three representative encoders—FLAVA (facebook/flava-full), ViT (google/vit-base-patch16-224), and CLIP (openai/clip-vit-base-patch32) [10]—on the same edge devices, as shown in Figure 1b. The RTX 4090 achieved the fastest performance, while the Jetson AGX Orin (NVIDIA, Santa Clara, CA, USA) and Orin Nano also completed encoding tasks within 1 s, indicating that lightweight encoding is feasible even on resource-constrained platforms.

These observations highlight a broader challenge in edge deployment: device heterogeneity. Edge environments typically consist of devices with varying computational capacities, memory sizes, and communication bandwidths. Several works have explored methods to improve performance in heterogeneous edge environments. EdgeFlow [11] introduces a progressive partitioning strategy for deep neural networks, distributing model layers across multiple edge devices to reduce inference latency. This DAG-based framework enables fine-grained model decomposition, achieving significant acceleration in distributed inference. In parallel, Liu et al. [12] propose a joint optimization framework that integrates task offloading with resource allocation, aiming to minimize system latency under dynamic edge conditions. Their approach models system constraints explicitly and leverages algorithmic coordination to improve responsiveness across devices. While these methods enhance performance in specific edge inference or offloading settings, they mainly target single-task pipelines and do not address the coordination of interdependent subtasks within multimodal workloads. Moreover, they overlook the economic objectives of real-world service providers. In practice, providers must balance task latency, resource utilization, and operational profit when scheduling MLLM workloads across heterogeneous edge devices. This makes profit-oriented scheduling not only desirable but essential for sustainable edge deployment.

Recent efforts have explored deploying LLMs in edge or edge–cloud collaborative systems to enhance responsiveness and resource efficiency. He et al. [13] propose an active inference-based offloading mechanism that improves the adaptability of LLM deployment without relying on explicit reward signals. By replacing traditional DRL objectives with task-specific inference guidance, their method achieves better generalization under varying workloads. In a more comprehensive setting, Huang et al. [14] design a two-timescale optimization framework for MLLM deployment and scheduling in edge–cloud environments. Their approach combines hierarchical reinforcement learning with attention and memory modules to jointly optimize model placement, GPU provisioning, and resource allocation across space and time. Both works demonstrate the potential of learning-based strategies for managing LLM workloads, but they rely on centralized architectures or domain-specific assumptions, and do not explicitly address the challenges of disaggregated MLLM execution under multi-provider, profit-driven constraints.

**Figure 2:**
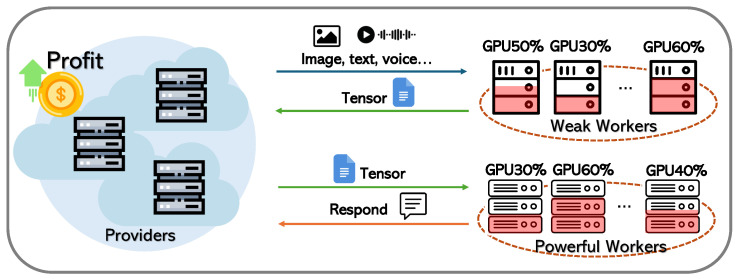
DistMLLM for multimodal large language model service.

To address these challenges, we propose DistMLLM, a novel framework designed to optimize the performance of MLLM services in heterogeneous edge environments. As illustrated in Figure 2, service providers first disaggregate multimodal language tasks and transmit raw multimodal data to less powerful edge devices, which are better suited for handling data-intensive operations. These devices perform multimodal encoding using lightweight GPUs and return the encoded tensor representations to the providers. The compact tensors are then forwarded to more powerful edge nodes for LLM inference. Finally, the inference results are sent back to the providers. This disaggregated execution strategy exploits the complementary strengths of heterogeneous devices while mitigating their individual limitations.

Efficient task allocation in such environments remains challenging due to unpredictable device loads and the conflicting interests of multiple service providers [15]. Multi-agent bandit algorithms have been widely used for decentralized task dispatch in uncertain and dynamic environments. For example, Chen et al. [16] propose $Crowd^{2}$, a bandit-based mechanism for assigning video analytics tasks in crowdsourcing platforms, which improves social welfare while maintaining fairness and sub-linear regret. Inspired by such approaches, DistMLLM incorporates a multi-agent bandit mechanism that operates across two layers: (i) an online learning layer that continuously estimates the utility of executing encoding and inference tasks on heterogeneous workers, and (ii) an allocation layer that performs coordinated task assignment based on these learned utilities. This design enables dynamic adaptation to real-time performance fluctuations and competing demands, ultimately maximizing provider profits while meeting task delay constraints.

Simulation results show that DistMLLM achieves the highest long-term profit and the lowest cumulative regret for both LLM and encoder tasks, consistently outperforming heuristic and UCB-based baselines. Its structured exploration enables faster convergence to high-reward assignments, while coordinated task allocation improves efficiency in dynamic and heterogeneous environments. DistMLLM also demonstrates strong scalability and robustness to transmission cost, with its disaggregated design yielding clear performance gains over monolithic execution.

## 2. Materials and Methods

### 2.1. Problem Formulation

In this section, we propose an online algorithm for task allocation in MLLM service providers. We aim to maximize the profit for all MLLM service providers while ensuring fairness and flexibility in task allocation. Table 2 summarizes the key notations used in the problem formulation.

We denote the set of MLLM service providers as M={1,2,...,M} and the set of edge workers as N={1,2,...,N}. The time is divided into discrete time slots T={1,2,...,T}. In our system, $x_{m,t}^{enc}$ represents the edge worker selected by MLLM service provider *m* to process the multimodal encoder at time slot *t*, and $x_{m,t}^{llm}$ represents the edge worker selected to process the large language model at time slot *t*.

The latency of the multimodal encoder and large language model inference performed by edge worker *n* when chosen by service provider *m* is denoted as $l_{m,n}^{enc}$ and $l_{m,n}^{llm}$, respectively. The energy consumption for these tasks is denoted as $e_{m,n}^{enc}$ and $e_{m,n}^{llm}$. The reward for service provider *m* when choosing edge worker *n* for these tasks is denoted as $r_{m,n}^{enc}$ and $r_{m,n}^{llm}$. Additionally, $c_{m}$ represents the computation demand of service provider *m* in each time slot, and $C_{n}$ represents the computing capacity of edge worker *n* in each time slot.

The latency of the multimodal encoder and large language model inference depends on the input data size $d_{m}$ and the device performance $p_{n}$ of the edge worker. The latency models are given by:

(1) $$l_{m,n}^{enc}=\psi_{n}^{enc}\left(d_{m}\right)\beta_{n}\left(p_{n}\right),$$

(2) $$l_{m,n}^{llm}=\psi_{n}^{llm}\left(d_{m}\right)\beta_{n}\left(p_{n}\right),$$

where the functions $\psi_{n}^{enc}\left(d\right)$ and $\psi_{n}^{llm}\left(d\right)$ represent the relationship between input data size and device performance with latency, respectively. These functions are concave.

Energy consumption mainly includes transmission and processing energy. The energy consumption models are given by:

(3) $$e_{m,n}^{enc}=\left(\gamma^{enc}+\mu \right)d_{m}\alpha {\left(p_{n}\right)}^{2},$$

(4) $$e_{m,n}^{llm}=\left(\gamma^{llm}+\mu \right)d_{m}\alpha {\left(p_{n}\right)}^{2},$$

where $\alpha {\left(p_{n}\right)}^{2}$ represents the device performance factor, and $\gamma^{enc}$, $\gamma^{llm}$, and μ represent the energy consumption for transmitting and processing one bit of data, respectively.

The reward for service provider *m* depends on the intrinsic value of the task, the latency of the multimodal encoder and large language model inference, and the energy consumption. The reward models are given by:

(5) $$r_{m,n}^{enc}=V\left(m\right)+G_{m}^{enc}\left(l_{m,n}^{enc}\right)-\omega_{n}e_{m,n}^{enc},$$

(6) $$r_{m,n}^{llm}=V\left(m\right)+G_{m}^{llm}\left(l_{m,n}^{llm}\right)-\omega_{n}e_{m,n}^{llm},$$

where V(m) represents the intrinsic value of the task, $G_{m}^{enc}\left(l\right)$ and $G_{m}^{llm}\left(l\right)$ are concave reward functions concerning latency, and $\omega_{n}$ represents the payment priced by edge worker *n* for consuming each unit of energy.

The optimization objective is to maximize the profit for all MLLM service providers, which can be represented as:

(7) $$P:\max_{x_{t}^{enc},x_{t}^{llm}\in T}\sum_{t\in T}\sum_{m\in M}\left(E\left[r_{m,x_{m,t}^{enc}}^{enc}\left(t\right)\right]+E\left[r_{m,x_{m,t}^{llm}}^{llm}\left(t\right)\right]\right),$$

subject to:

(8) $$\sum_{m\in M_{n,t}}c_{m}\leq C_{n},\forall n\in N,\forall t\in T.$$

In time slot *t*, a decision $x_{t}^{*}=\left(x_{m,t}^{*}\right)$ has the property of proportional fairness if, for any other feasible decision $x_{t}^{\prime }=\left(x_{m,t}^{\prime }\right)$, the following inequality holds:

(9) $$\sum_{m\in M}\frac{r_{m,x_{m,t}^{\prime }}-r_{m,x_{m,t}^{*}}}{r_{m,x_{m,t}^{*}}}\leq 0.$$

### 2.2. Algorithm Design

To tackle the stochastic changes in rewards, we design a online algorithm based on a multi-agent bandit approach. This algorithm explores the potential rewards from different edge workers and exploits the best-known configurations.

The proposed algorithm follows these detailed steps: First, initialize the performance data for each edge worker and set the parameters for exploration and exploitation. In the exploration phase, each MLLM service provider randomly allocates both the multimodal encoder and the large language model tasks to different edge workers for a predefined number of rounds. The performance estimates are updated based on the observed latency and energy consumption. Next, the multi-knapsack problem solver is called to determine the optimal allocation mapping in the exploitation phase. Tasks are dispatched based on this optimal allocation mapping for a predefined number of exploitation rounds. Throughout this phase, the performance estimates are continuously updated to adapt to any changes in edge worker performance.

### 2.3. Mapping upon Multi-Knapsack Problem

To obtain the optimal task allocation mapping Π, we model it as a multi-knapsack problem for each time slot *t*. The objective is to maximize the utility function, which combines the performance of the multimodal encoder and the large language model tasks.

(10) $$\max_{x_{t}}\sum_{m\in M}\left(u_{\tau m,x_{m,t}^{enc}}^{enc}+u_{\tau m,x_{m,t}^{llm}}^{llm}\right),$$

subject to:

(11) $$\sum_{m\in M_{n,t}}c_{m}\leq C_{n},\forall n\in N.$$

The mapping algorithm is designed to solve this multi-knapsack problem iteratively, updating the allocation mapping Π based on the observed performance.

The regret in our algorithm is defined as the difference between the reward achieved by the optimal allocation and the reward obtained by our algorithm. The proposed algorithm balances exploration and exploitation effectively to minimize regret. The regret can be quantified as follows:

(12) $$R\left(T\right)=\sum_{t=1}^{T}\sum_{m\in M}\left(r_{m,x_{m,t}^{*}}-r_{m,x_{m,t}}\right),$$

where $r_{m,x_{m,t}^{*}}$ is the reward for the optimal allocation and $r_{m,x_{m,t}}$ is the reward achieved by our algorithm at time *t*. The algorithm aims to reduce this regret over time by alternating between exploration and exploitation.

Exploration and exploitation are critical components of our algorithm. During exploration, MLLM service providers gather information about the performance of various edge workers by randomly allocating tasks. This phase is essential to build an accurate edge workers’ capabilities model. In each epoch, the exploration phase uses a fixed exploration time $T_{\mathrm{explore}}$ to gather sufficient data. The estimated reward ${\hat{u}}_{m,n}^{enc}$ is initialized as 0 and updated as:

(13) $${\hat{u}}_{m,n}^{enc}=\left({\hat{u}}_{m,n}^{enc}\times \left(\tau -1\right)+u_{m,n}^{enc}\right)/\tau ,$$

(14) $${\hat{u}}_{m,n}^{llm}=\left({\hat{u}}_{m,n}^{llm}\times \left(\tau -1\right)+u_{m,n}^{llm}\right)/\tau ,$$

where τ is the current epoch. In the exploitation phase, the algorithm uses the gathered information to make informed decisions, allocating tasks to the best-performing edge workers to maximize rewards. By alternating between exploration and exploitation, our algorithm ensures that it remains adaptive and continues to optimize task allocations even as conditions change.

### 2.4. Algorithm Analysis

This section analyzes the proposed online task allocation algorithm. We provide high-probability estimation accuracy bounds, a robustness condition under which the mapping is invariant to estimation error, a characterization of the multi-knapsack subroutine based on branch-and-bound (B&B), and a logarithmic regret bound.

Unless otherwise stated, rewards are assumed to be uniformly bounded. For each provider–worker pair (m,n), let $u_{m,n}^{enc}\in \left[u_{\min},u_{\max}\right],$ where $u_{\max}:=\max_{m,n}u_{m,n}^{enc}$ and $u_{\min}:=\min_{m,n}u_{m,n}^{enc}$ denote the maximal and minimal feasible utility values across all assignments. Thus,

(15) $$u_{\min}\leq u_{m,n}^{enc}\leq u_{\max},\;R:=u_{\max}-u_{\min}<\infty ,$$

capacities satisfy $C_{n}>0$ for each worker n∈N, and item costs satisfy $c_{m}\geq c_{\min}>0$ for each service provider m∈M. We denote

(16) $$N_{\max}:=\max_{n\in N}\left\lfloor \frac{C_{n}}{c_{\min}}\right\rfloor ,\;B:=\sum_{n\in N}\left\lfloor \frac{C_{n}}{c_{\min}}\right\rfloor \left(\leq NN_{\max}\right).$$

Algorithm 1 proceeds in epochs τ=1,2,…. Each epoch consists of (i) an exploration phase of $T_{\mathrm{explore}}$ slots and (ii) an exploitation phase of length $2^{\tau }$ where Algorithm 2 (the multi-knapsack subroutine) is invoked using current estimates $\left\{{\hat{u}}_{m,n}^{enc}\right\}$. After τ epochs, each pair (m,n) has accumulated at least τ i.i.d. reward samples. The LLM side is symmetric and omitted for brevity.
**Algorithm 1** Task Allocation with Multi-Agent Bandit  1:**Input:** exploration length $T_{\mathrm{explore}}$, exploitation length $T_{\mathrm{exploit}}=2^{\tau }$  2:Initialize $u_{\tau m,n}^{enc}\leftarrow 0$, $u_{\tau m,n}^{llm}\leftarrow 0$, ∀m∈M,∀n∈N  3:**for** τ=1 to $\tau_{T}$ **do**  4:    **for** t=1 to $T_{\mathrm{explore}}$ **do**  5:        **for** each service provider m∈M **do**  6:           Each provider *m* selects a worker n∈N in a round-robin order and sends encoder and LLM subtasks, observing rewards $u_{m,n}^{enc}\left(\tau \right)$ and $u_{m,n}^{llm}\left(\tau \right)$  7:           Update $u_{\tau m,n}^{enc}\leftarrow \left(u_{(\tau -1)m,n}^{enc}\times \left(\tau -1\right)+u_{m,n}^{enc}\left(\tau \right)\right)/\tau$  8:           Update $u_{\tau m,n}^{llm}\leftarrow \left(u_{(\tau -1)m,n}^{llm}\times \left(\tau -1\right)+u_{m,n}^{llm}\left(\tau \right)\right)/\tau$  9:        **end for**10:    **end for**11:    **Call** Algorithm 2 with input $\left\{u_{\tau m,n}^{enc},u_{\tau m,n}^{llm}\right\}$ and Π=012:    **for** t=1 to $T_{\mathrm{exploit}}$ **do**13:        Service providers dispatch MLLM inference tasks based on Π14:    **end for**15:**end for**

**Algorithm 2** Multi-Knapsack Mapping
  1:**Input:**$\left\{u_{\tau m,n}^{enc},u_{\tau m,n}^{llm}\right\}$, Π=0  2:**for** each edge worker n∈N **do**  3:    **for** each service provider m∈M **do**  4:        **if** $\Pi_{m}\neq 0$ **then**  5:            $\Delta u_{\tau m,n}^{enc}\leftarrow u_{\tau m,n}^{enc}-u_{\tau m,\Pi_{m}}^{enc}$  6:            $\Delta u_{\tau m,n}^{llm}\leftarrow u_{\tau m,n}^{llm}-u_{\tau m,\Pi_{m}}^{llm}$  7:        **else**  8:            $\Delta u_{\tau m,n}^{enc}\leftarrow u_{\tau m,n}^{enc}$  9:            $\Delta u_{\tau m,n}^{llm}\leftarrow u_{\tau m,n}^{llm}$10:        **end if**11:    **end for**12:    Solve the knapsack sub-problem for worker *n* with $\Delta u_{\tau m,n}^{enc}$ and $\Delta u_{\tau m,n}^{llm}$13:    **for** each service provider m∈M **do**14:        **if** worker *n* accepts task from provider *m* **then**15:           Update $\Pi_{m}\leftarrow n$16:        **end if**17:    **end for**18:
**end for**
19:
**Output:** Π

**Lemma** **1.** *After τ independent samples for pair (m,n), for any ϵ>0,*

(17) $$\mathrm{Pr}\left(|{\hat{u}}_{m,n}^{enc}-u_{m,n}^{enc}|>\epsilon \right)\leq 2\exp\left(-\frac{2\tau \epsilon^{2}}{R^{2}}\right).$$

*Consequently, for the collection of all M×N pairs,*

(18) $$\mathrm{Pr}\left(\exists \left(m,n\right):|{\hat{u}}_{m,n}^{enc}-u_{m,n}^{enc}|>\epsilon \right)\leq 2MN\exp\left(-\frac{2\tau \epsilon^{2}}{R^{2}}\right).$$

**Proof.**  Apply Hoeffding’s inequality to τ i.i.d. samples supported on an interval of width *R*. A union bound over M×N pairs yields the second inequality. □

**Remark** **1.** *For any target failure level $\eta_{\tau }\in \left(0,1\right)$ at epoch τ, one can choose the number of exploration samples per pair (m,n) large enough so that*

(19) $$2MN\exp\left(-\frac{2\tau \epsilon_{\tau }^{2}}{R^{2}}\right)\leq \eta_{\tau },$$

*where $\epsilon_{\tau }$ is the desired accuracy in epoch τ. In practice, this can be enforced by letting each pair (m,n) obtain at least one additional sample per epoch, so that after τ epochs each pair has at least τ samples, and by decreasing $\epsilon_{\tau }$ as τ increases.*

**Lemma** **2.** *Let $\Pi^{★}$ be the optimal mapping under true rewards with total value $V(\Pi^{★})$. Define the optimality gap*

(20) $$\Delta_{\mathrm{gap}}:=\min_{\Pi \neq \Pi^{★}}\left(V\left(\Pi^{★}\right)-V\left(\Pi \right)\right)\left(>0\right).$$

*If $\max_{m,n}|{\hat{u}}_{m,n}^{enc}-u_{m,n}^{enc}|\leq \varepsilon$ and $\varepsilon \leq \Delta_{\mathrm{gap}}/\left(2B\right)$, then the mapping $\hat{\Pi }$ derived from $\left\{{\hat{u}}_{m,n}^{enc}\right\}$ coincides with $\Pi^{★}$.*

**Proof.**  Any feasible mapping contains at most *B* assignments. Entrywise perturbations bounded by ε change any mapping’s total value by at most Bε. Hence the estimated advantage of $\Pi^{★}$ over any $\Pi \neq \Pi^{★}$ is at least $\Delta_{\mathrm{gap}}-2B\varepsilon \geq 0$, preserving optimality. □

We instantiate Algorithm 2 as a B&B solver for each multi-knapsack subproblem constructed from the current estimates $\left\{{\hat{u}}_{m,n}^{enc}\right\}$. When fully executed, B&B is exact; with time or node limits, it behaves as an anytime heuristic with a certified optimality gap.

**Lemma** **3.** *When executed to completion (without time/node limits), the B&B solver returns the global optimum of the multi-knapsack subproblem. Its worst-case running time is exponential. Under time- or node-limited execution, B&B becomes an anytime heuristic that returns a feasible incumbent along with a valid upper bound, thereby certifying an optimality gap at termination; no fixed constant-factor approximation ratio is guaranteed.*

**Proof.**  When executed without time or node limits, B&B explores a search tree where each node carries a valid upper bound and the algorithm maintains a feasible incumbent solution. Since pruning only removes nodes whose upper bound is no larger than the incumbent, full exploration guarantees that no optimal solution is discarded. Hence, B&B returns the exact global optimum when allowed to run to completion, although its worst-case complexity is exponential due to the NP-hardness of the multi-knapsack problem.

When terminated early, B&B functions as an anytime heuristic. At termination, it retains a feasible incumbent of value *L* and a valid upper bound *U*, and therefore provides a certified optimality gap U−L. Because the incumbent may lie anywhere in the search tree, no instance-independent constant-factor approximation ratio can be guaranteed under arbitrary time limits. □

**Theorem** **1.** *Let Algorithm 1 run for T slots with the epoch-based schedule described above and Algorithm 2 instantiated by the B&B solver as in Lemma 3. Then, for the ENC pipeline, the cumulative regret is bounded by*

(21) $$R_{\mathrm{ENC}}\left(T\right)\leq \left(T_{\mathrm{explore}}+M\right)Nu_{\max}\log_{2}\left(T+2\right)+C_{0}=O\left(\log T\right),$$

*where $C_{0}=O\left(N^{2}Mu_{\max}\right)$ is a constant independent of T. An identical bound holds for the LLM pipeline, i.e., $R_{\mathrm{LLM}}\left(T\right)=O\left(\log T\right)$.*

**Proof.**  Let $\tau_{T}$ be the last completed epoch by time *T*. Since the exploitation length of the τ-th epoch equals $2^{\tau }$, we have

(22) $$T\geq \sum_{\tau =1}^{\tau_{T}}2^{\tau }=2\left(2^{\tau_{T}}-1\right)\Rightarrow \tau_{T}\leq \log_{2}\left(T+2\right).$$

We decompose the regret into three components.

*(i) Exploration.* During exploration, each provider collects $T_{\mathrm{explore}}$ samples per epoch, and the reward is at most $u_{\max}$ for each provider–worker pair. Thus, the regret contributed during exploration is at most $\left(T_{\mathrm{explore}}Nu_{\max}\right)\tau_{T}$.

*(ii) Solver suboptimality.* When B&B is run without time limits, the multi-knapsack subproblem is solved optimally and this term becomes zero. Under practical time budgets, B&B returns a feasible incumbent with a certified optimality gap, and the loss per epoch remains uniformly bounded. Therefore, the cumulative contribution of this term is absorbed into the constant $C_{0}$.

*(iii) Estimation errors.* Let $E_{\tau }$ denote the event that the uniform estimation bound in Lemma 1 fails at epoch τ. The sampling schedule ensures $\mathrm{Pr}\left(E_{\tau }\right)\leq \eta_{\tau }$, where $\eta_{\tau }$ decays exponentially. The worst-case regret in epoch τ is $O(2^{\tau }NMu_{\max})$, and the expected regret is thus $O(2^{\tau }NMu_{\max}\eta_{\tau })$. Selecting $\eta_{\tau }\leq 2^{-2\tau }$ ensures that $\sum_{\tau \geq 1}2^{\tau }\eta_{\tau }$ converges, introducing only the constant term $C_{0}$.

Combining the above three parts and using $\tau_{T}\leq \log_{2}\left(T+2\right)$ yields the claimed O(logT) bound. □

**Remark** **2** (On the exploration length $T_{\mathrm{explore}}$)**.** *The length of the exploration phase arises from the requirement that all NM provider–worker utilities be estimated with uniform accuracy at the beginning of each epoch. According to Lemma 1, obtaining estimates within accuracy $\epsilon_{\tau }$ and failure probability at most $\eta_{\tau }$ requires each provider–worker pair to be sampled a sufficient number of times. This naturally introduces factors involving N, M, and $u_{\max}$ in $T_{\mathrm{explore}}$. These constants are a consequence of standard concentration inequalities and do not reflect computational overhead; hence, they do not affect the real-time execution or scalability of the algorithm.*

## 3. Results

In this section, we first present a set of preliminary device-level experiments, followed by extensive simulations that demonstrate the effectiveness of the proposed algorithm compared with existing alternatives.

### 3.1. Preliminary Experiment

We first conduct a preliminary device-level experiment that characterizes both the communication cost of encoder outputs and the latency composition of heterogeneous execution.

**Figure 3:**
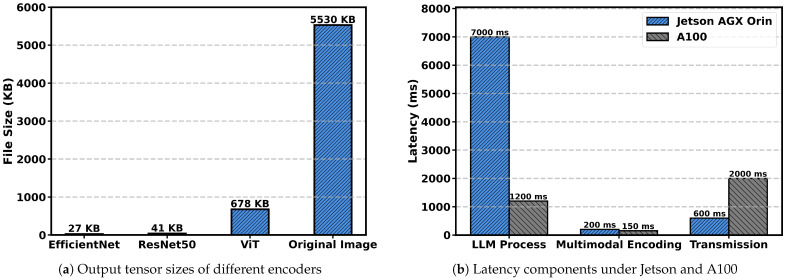
Comparison of encoder tensor sizes and latency breakdowns. (**a**) Output tensor sizes for different visual encoders. (**b**) Latency components of LLM processing, multimodal encoding, and transmission under Jetson AGX Orin and A100.

We begin by assessing the communication benefit brought by multimodal encoders. A 4K-resolution (3840 × 2160) PNG image of approximately 5.4 MB is used as the representative input. As shown in Figure 3a, the output tensors generated by several visual encoders are significantly smaller than the original image, with lightweight backbones such as EfficientNet and ResNet50 achieving more than an order-of-magnitude reduction.

Next, we analyze the end-to-end latency using two computing platforms: a Jetson AGX Orin (64 GB) representing the low-power edge device, and a remote NVIDIA A100 (NVIDIA, Santa Clara, CA, USA) representing high-performance edge device. We adopt the LLaVA-1.5-7B model as a representative MLLM and measure the latency contributions from multimodal encoding, LLM inference, and data transmission. Figure 3b illustrates the latency decomposition under the two execution settings. For Jetson AGX Orin, the majority of the delay originates from LLM processing due to the limited computational capability of embedded hardware. In contrast, remote execution significantly accelerates LLM inference on the A100, but this performance advantage is offset by substantial transmission delay caused by sending large raw inputs to the server.

Overall, these results reveal a clear performance trade-off within heterogeneous edge environments. Lightweight edge devices can efficiently perform multimodal encoding and produce compact feature tensors, but they are unable to execute LLM inference with acceptable latency due to limited computational capability. Conversely, although powerful edge nodes can perform LLM inference with low latency, such nodes are typically scarce and often geographically distant from end devices, making it inefficient to transmit large raw inputs directly to them.

### 3.2. Simulation Experiment Settings

Building on the above device-level observations, we now conduct large-scale simulations to systematically evaluate how task allocation strategies behave under diverse provider and worker configurations. We use synthetic datasets for providers to simulate large language model and encoder tasks dispatched to workers. Each worker is modeled with a random performance metric drawn from a uniform distribution between 0.8 and 1.0. The number of time slots for the simulation is fixed at 500. To ensure statistical robustness, each simulation scenario is repeated 20 times, and we report the mean performance along with its standard deviation. We test different system scales by varying both the number of providers and the number of workers. Specifically, the number of providers is set to {5, 6, 7}, while the number of workers is varied as {5, 10, 20, 30}.

For the algorithms, we compare our DistMLLM algorithm with two baselines. HEU (heuristic baseline): a simple two-tier heuristic that assigns ENC tasks to weaker workers and LLM tasks to stronger workers based on their averaged performance ranks. UCB (multi-agent UCB) [17]: a decentralized UCB-based method in which each provider independently runs a UCB policy for both ENC and LLM subtasks.

### 3.3. Profit and Regret Analysis

**Figure 4:**
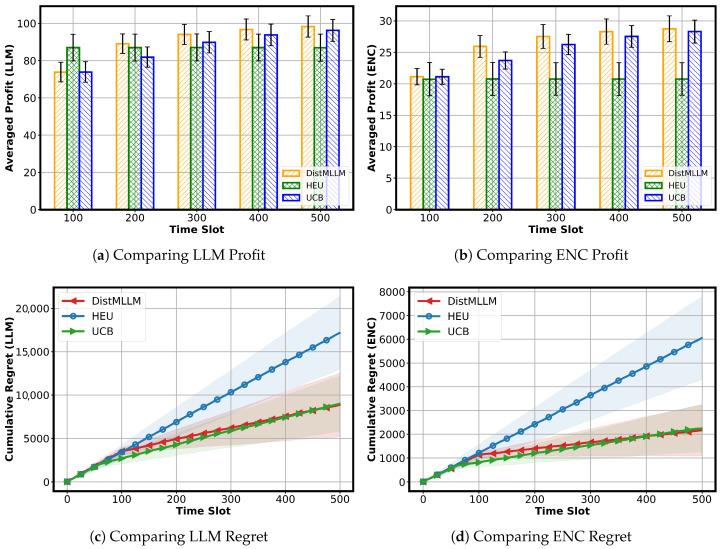
Performance comparison of different algorithms for LLM and ENC tasks (error bars and shaded areas indicate the standard deviation over 20 runs).

**Comparing LLM Profit:** We first examine the averaged profit of LLM tasks, as shown in Figure 4a. At the beginning of the simulation (approximately the first 100 time slots), DistMLLM undergoes an explicit exploration phase, resulting in lower LLM profit compared with HEU. Since HEU follows a fixed assignment rule without any exploration cost, it initially achieves the highest profit among the three algorithms. However, once DistMLLM completes its exploration stage and enters stable exploitation, it quickly converges to a superior assignment strategy. After this transition, DistMLLM consistently outperforms both HEU and UCB for the remainder of the simulation, maintaining the highest time-averaged LLM profit.

The HEU baseline attains moderate performance by assigning LLM tasks to stronger workers based on averaged performance ranks. However, its static allocation prevents it from adapting to dynamic performance variations across workers. The UCB algorithm yields the lowest profit, as its decentralized exploration strategy causes each provider to independently explore the worker space, incurring substantial exploration overhead. These results demonstrate that DistMLLM more effectively leverages cross-layer cooperation to optimize LLM execution.

We next examine the time-averaged profit of ENC tasks, as shown in Figure 4b. Although the absolute profit scale of ENC tasks is lower than that of LLM tasks, the three algorithms still exhibit a clear performance hierarchy. DistMLLM consistently achieves the highest ENC profit at all observed time points and continues to improve as the simulation progresses, indicating that its coordinated cross-layer scheduling also benefits the encoder side. UCB attains the second-best performance, with its profit steadily increasing but remaining below that of DistMLLM. In contrast, the HEU baseline yields the lowest ENC profit and remains almost flat over time, because its static split of workers into ENC tier and LLM tier cannot fully exploit worker heterogeneity or adapt to dynamic performance fluctuations. These results show that, even for relatively lightweight ENC tasks, adaptive multi-agent learning still brings a noticeable advantage over fixed heuristic assignment.

**Comparing LLM and ENC Regret:** We further examine the cumulative regret of the three algorithms for both LLM and ENC tasks, as shown in Figure 4c,d. Since regret characterizes the performance loss with respect to the oracle optimum, lower curves indicate more efficient online learning.

For LLM tasks, HEU suffers from the highest cumulative regret with a consistently steep growth rate, reflecting the persistent suboptimality of its static worker assignment. During the early and mid stages (roughly the first 400 time slots), DistMLLM incurs slightly higher regret than UCB due to its coordinated exploration. However, as learning progresses, DistMLLM gradually reduces its regret growth rate and eventually surpasses UCB, ending the simulation with the lowest overall LLM regret.

For ENC tasks, HEU again exhibits the largest cumulative regret. In the earlier stage of the simulation, UCB maintains the lowest regret among the three algorithms, slightly lower than DistMLLM. As time progresses, however, DistMLLM continues to refine its ENC–LLM joint assignment strategy and ultimately overtakes UCB in the final portion of the horizon, finishing with the lowest ENC regret overall. This demonstrates that although ENC tasks are lighter and more stable, cross-layer coordination still provides long-term advantages, allowing DistMLLM to achieve superior regret performance for both LLM and ENC tasks.

**Figure 5:**
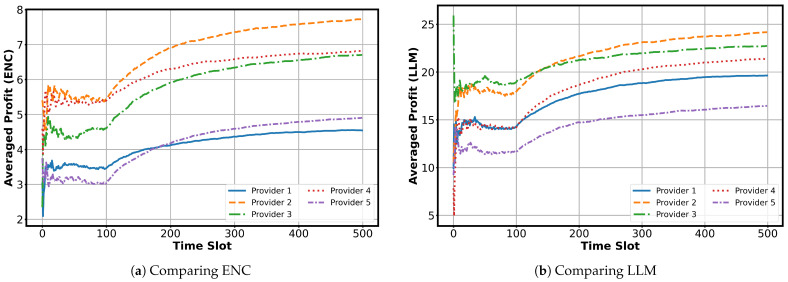
Averaged ENC and LLM profit of individual providers under the DistMLLM algorithm.

**Provider-Level Performance under DistMLLM:** Finally, Figure 5 illustrates the time-averaged ENC and LLM profit of the five providers under the DistMLLM algorithm. At the beginning of the simulation, DistMLLM conducts explicit exploration by cycling each provider across different workers. Because these assignments are not guided by reward estimates, the provider-level profits exhibit noticeable fluctuations.

After exploration concludes, the algorithm switches to exploitation. It constructs an allocation mapping using the performance estimates gathered so far and maintains this mapping for an extended period. From this point onward, the profit curves stabilize and gradually converge. Providers that consistently match well with high-performing workers achieve higher long-term averages, while others converge to lower levels based on their estimated utility profiles.

A clear contrast emerges between ENC and LLM results. ENC profits show relatively small differences among providers, which is consistent with their lower task value and weaker sensitivity to worker heterogeneity. In comparison, LLM profits exhibit a wider spread because LLM tasks rely more heavily on worker performance and carry higher intrinsic value. As a result, variations in worker suitability translate more strongly into provider-level differences during convergence.

Across all experiments in this section, DistMLLM demonstrates clear and consistent advantages over both HEU and UCB in terms of profit and regret. For LLM and ENC tasks alike, DistMLLM quickly transitions from exploration to stable exploitation and achieves the highest long-term averaged profit. The regret results further reinforce this observation: although DistMLLM may incur slightly higher regret in the early stage due to its structured exploration, it ultimately surpasses UCB and ends with the lowest cumulative regret in both task types.

### 3.4. Sensitivity Analysis

**Figure 6:**
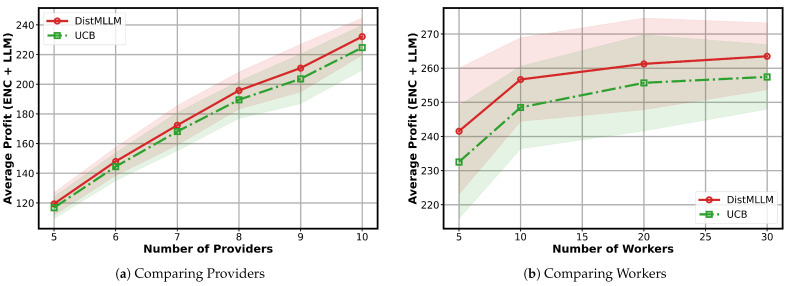
Analysis of MLLM task performance across providers and workers (shaded areas indicate the standard deviation over 20 runs).

**Figure 7:**
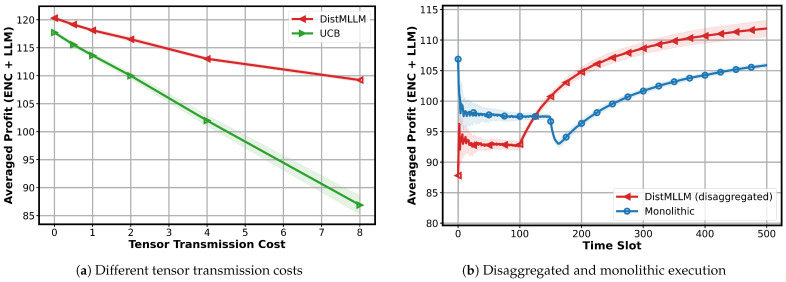
Evaluating the effect of disaggregation and tensor transmission cost (shaded areas indicate the standard deviation over 20 runs).

Beyond the main comparison with baseline algorithms, we conduct a set of ablation and sensitivity experiments to further examine how different system factors influence the performance of DistMLLM. These experiments evaluate (i) scalability with respect to the number of service providers and workers, (ii) the benefit brought by disaggregation, and (iii) the impact of tensor transmission cost. The results are summarized in Figure 6 and Figure 7.

**Impact of the number of providers and workers.**   Figure 6a shows the averaged profit as the number of service providers increases from {5, 6, 7, 8, 9, 10}, while the number of workers is fixed at 5. Across all settings, DistMLLM consistently outperforms the decentralized multi-agent UCB baseline. As the number of providers increases under this fixed worker budget, the system becomes progressively congested, and the independent exploration conducted by UCB leads to more frequent collisions and inefficient worker utilization. In contrast, DistMLLM leverages its coordinated exploration–exploitation mechanism to mitigate contention and maintain more efficient worker allocation, resulting in significantly higher total profit and demonstrating strong scalability with respect to provider population.

**Impact of the number of providers and workers.**   Figure 6a shows the averaged profit as the number of service providers increases from {5, 6, 7, 8, 9, 10}, while the number of workers is fixed at 5. Across all settings, DistMLLM consistently outperforms the decentralized multi-agent UCB baseline. As the number of providers increases under this fixed worker budget, the system becomes progressively congested, and the independent exploration conducted by UCB leads to more frequent collisions and inefficient worker utilization. In contrast, DistMLLM leverages its coordinated exploration–exploitation mechanism to mitigate contention and maintain more efficient worker allocation, resulting in significantly higher total profit and demonstrating strong scalability with respect to provider population.

Figure 6b shows the averaged profit as the number of workers varies over {5, 10, 20, 30}, while the number of service providers is fixed at 10. As more workers become available, both algorithms initially benefit from the increased computational capacity; however, DistMLLM exhibits a noticeably larger performance improvement. When the worker pool is small, contention among providers is high, and the decentralized exploration conducted by the multi-agent UCB baseline leads to frequent conflicts and inefficient worker allocation.

A notable observation is that, after reaching the peak at around 10 workers, the performance of both algorithms slightly decreases as the worker pool continues to expand. This decline occurs because excessively large worker pools dilute the effective exploration signals: each provider requires more time to sample all available workers, causing slower convergence to high-reward assignments. Additionally, when many low-quality workers are introduced, the probability of sampling suboptimal workers increases, temporarily dragging down the averaged total profit. Despite this downward trend at large worker counts, DistMLLM remains consistently superior to UCB.

**Impact of tensor transmission cost and disaggregation.**   Figure 7a evaluates how varying the communication cost between encoder and LLM components affects performance. As the transmission cost increases, the profit of both algorithms decreases, which is expected since offloading encoders and LLMs to different workers becomes less attractive. However, DistMLLM exhibits a much slower degradation curve, maintaining a consistent advantage over the UCB baseline. This demonstrates that the disaggregated decision mechanism of DistMLLM is more robust to communication overhead.

In addition, to isolate the benefit of MLLM disaggregation, Figure 7b compares DistMLLM with a monolithic version in which each provider must assign both the encoder and LLM to the same worker. The monolithic design initially achieves a stable profit but is unable to fully exploit worker heterogeneity. DistMLLM, after completing its exploration phase, rapidly converges to higher profit by independently placing the encoder and LLM on different workers when advantageous. This illustrates that decoupling the two components provides clear performance gains—especially in heterogeneous environments where worker strengths differ significantly across encoder and LLM tasks.

Overall, these results highlight the importance of coordinated exploration and joint encoder–LLM allocation in heterogeneous edge environments and confirm that disaggregation is a key enabler of efficient MLLM inference on the edge.

## 4. Conclusions

In conclusion, this work presents DistMLLM, a framework for deploying multimodal large language model services in heterogeneous edge environments through a disaggregated execution strategy. Firstly, by separating the encoding of multimodal data and the inference of LLM across lightweight and high-performance edge devices, DistMLLM leverages the complementary hardware capabilities of heterogeneous platforms. This significantly reduces end-to-end latency and transmission overhead by using compact tensor representations, enabling realistic deployment on resource-constrained edge nodes. Secondly, in order to address dynamic workloads and competing service provider interests, DistMLLM integrates a multi-agent bandit algorithm that continuously estimates device utilities and allocates tasks jointly across encoders and LLM workers. Thirdly, extensive experiments demonstrate that, by combining realistic device-level measurements and large-scale simulations, DistMLLM achieves higher long-term profit, lower cumulative regret and more stable performance than representative multi-agent UCB baselines and heuristic baseline. Furthermore, it shows strong robustness to variations in communication costs and heterogeneous device capabilities. These results highlight the promise of disaggregated execution coupled with adaptive bandit-based scheduling for practical MLLM deployment in real-world edge computing systems.

## Figures and Tables

**Table 1:** Summary of equipment parameters.

Devices Information				
Name	GPU	RAM	Storage	Power
Jetson Orin Nano	1024 CUDA	8 GB	microSD/SSD	5–15 W (Configurable)
Jetson AGX Orin	2048 CUDA	64 GB	NVMe SSD	15–60 W (Configurable)
RTX 4090	16,384 CUDA	24 GB	NVMe SSD	450 W

**Table 2:** List of notations.

Notation	Description
*M*	Set of MLLM service providers
*N*	Set of edge workers
*T*	Set of discrete time slots
$x_{m,t}$	Edge worker selected by service provider *m* at time slot *t*
$l_{m,n}$	Latency of the edge worker *n* when chosen by service
	provider *m*
$e_{m,n}$	Energy consumption for service provider *m* when choosing
	edge worker *n*
$r_{m,n}$	Reward for service provider *m* when choosing edge
	worker *n*
$c_{m}$	Computation demand of service provider *m* in each
	time slot
$C_{n}$	Computing capacity of edge worker *n* in each time slot
$d_{m}$	Input data size for service provider *m*
$p_{n}$	Device performance of edge worker *n*

## Data Availability

The data presented in this article are available upon request from the corresponding author.
